# Supplementary material for: A Role for iNOS in Erastin Mediated Reduction of P-Glycoprotein Transport Activity
Source: Cancers (Basel). 2024 Apr 29;16(9):1733. doi: 10.3390/cancers16091733 (PMC11083490; doi:10.3390/cancers16091733)
Supplement: Supplementary file 1 [file cancers-16-01733-s001.zip › cancers-2978745-supplementary.pdf]

Supplementary Figure 1A - Full Western Blots used in Figure 4 in main text

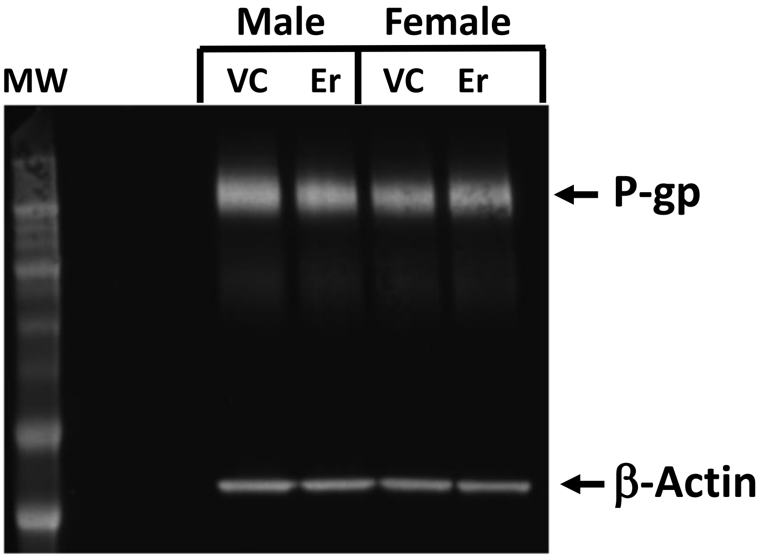

Qualitative Western of P-glycoprotein levels relative to beta-actin in male and female BBB after exposure to erastin.

Supplementary Figure 1B - Full Western Blots used in Figure 4 in main text

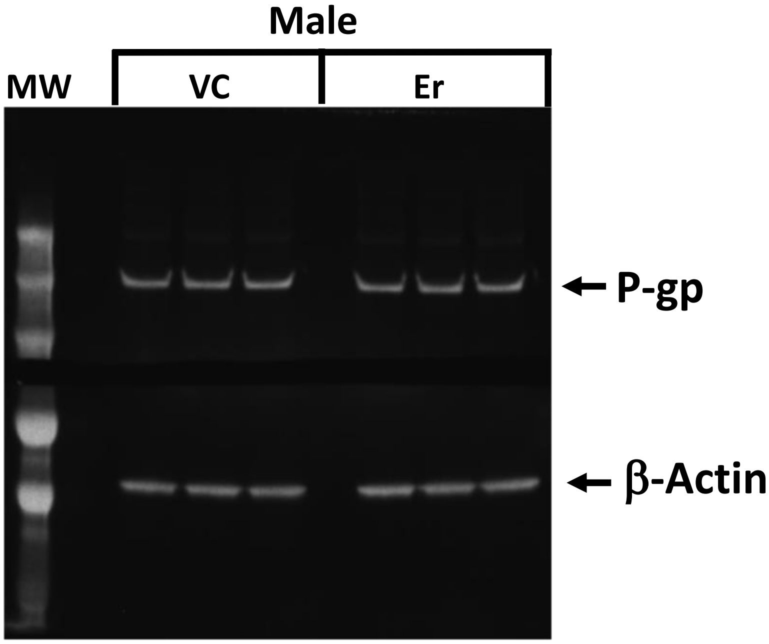

Quantitative western blot to examine the potential for differences in the levels of P-gp in male rat brain capillaries exposed to erastin relative to untreated controls.

Supplementary Figure S1C – Densitometry measurments of bands used in Figure 4C in main text

	VC			Er		
P-gp →	1657 x (1.03)	1662 x (1.03)	1786 x (1.0)	1673 x (1.0)	1659 x (1.1)	1683 x (1.13)
β-Actin →	2045	2050	2102	2147	1953	1902

Quantitation of western blot above: P-glycoprotein levels in male brain capillaries normalized ( ) to beta-actin levels after exposure to erastin.

Supplementary Figure 2 C and D.

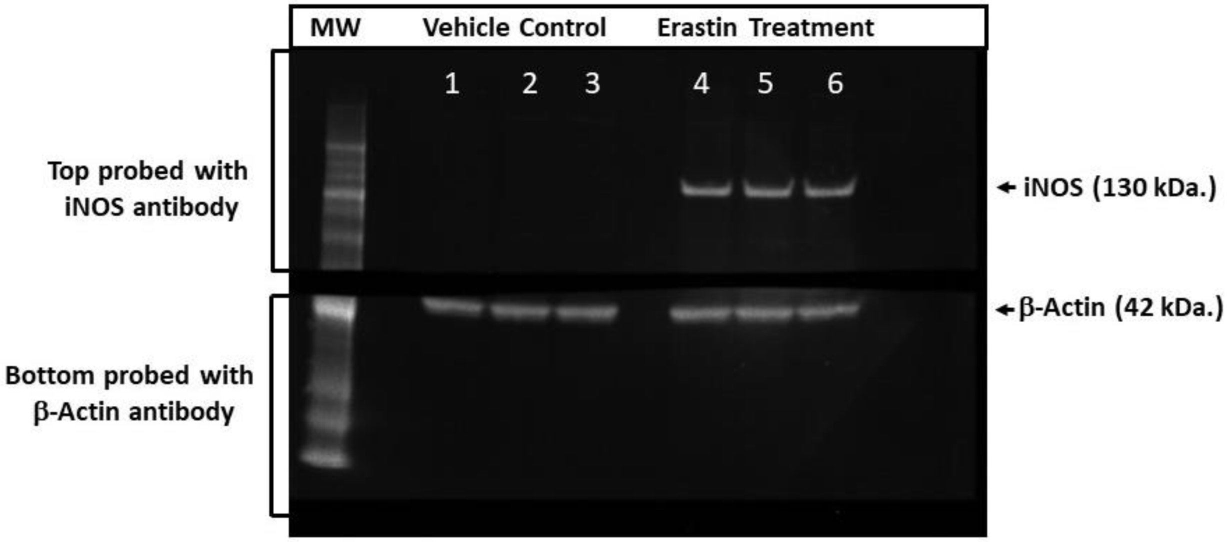

Western blot of iNOS (top) and β-Actin (bottom) protein levels measured in rat brain capillaries in triplicate. This blot was used for the **Figure 6 (C & D)** in the main text to quantitate levels of each respective protein.
